# Frequency of allogenic blood transfusion in patients with gastrointestinal cancer: a cross-sectional study in Peru

**DOI:** 10.3332/ecancer.2021.1289

**Published:** 2021-09-14

**Authors:** Jeel Moya-Salazar, Eulogio Cáceres, Jorgelina Blejer, Carlos Gonzalez, Hans Contreras-Pulache

**Affiliations:** 1 Pathology Department, Hospital Nacional Docente Madre-Niño San Bartolomé, Lima 15001, Peru; 2 School of Medicine, Faculties of Health Science, Universidad Norbert Wiener, Lima 15001, Peru; 3 Service of Blood Bank, Department of Clinical Pathology, Oncosalud, Lima 15001, Peru; 4 Transfusion Transmissible Infections Section, Fundación Hemocentro Buenos Aires, Buenos Aires 1407, Argentina; 5 Hemotherapy Department, Hospital de Infecciosas F.J. Muñiz, Buenos Aires 1407, Argentina

**Keywords:** gastrointestinal cancer, transfusion, blood products, blood bank, Peru

## Abstract

**Background:**

Gastrointestinal cancer demands a high frequency of transfusions, and the high availability of blood products. We aimed to determine the frequency of blood transfusions and the most used blood products according to the type of gastrointestinal cancer.

**Methods:**

A cross-sectional study was conducted in a Peruvian Type I Hemotherapy and Blood Bank Service of a Private Oncological Clinic during 2016–2018. We included patients with gastrointestinal cancer using the International Code of Diseases. The donations were made in compliance with the requirements of the Programa Nacional de Hemoterapía y Banco de Sangre and in accordance with the Standardised Operational Procedure of the clinic.

**Results:**

We analysed 3,022 patients, of which 163 (5.4%) had gastrointestinal cancer (67.1 ± 12 years). The 80 (49.1%) men did not show significant differences with the 83 (50.9%) women (*p* = 0.178). The most frequent neoplasia was the colon (41.7%) and pancreas (37.4%). Three hundred and four blood products were transfused (average 1.8 ± 2.5 units (range: 1–30 units/patient)), of which 81.3% (247 units) were red blood cells concentrated, 8.6% (26 units) were fresh-frozen-plasma (FFP) and 6.6% (20 units) were cryoprecipitate. The type of cancer that most blood products demanded was colon neoplasia (41.8%), followed by pancreatic cancer (26.3%) and liver cancer (10.9%). We determined that ~55% of patients were O Rh(D)+ and in five patients we were poly-transfused.

**Conclusion:**

Our findings suggested that patients with gastrointestinal cancer require large numbers of transfusions of blood cell concentrate and FFP. Also, we showed that cancer of the colon, pancreas and liver demanded more than 75% of blood products.

## Introduction

Following their anatomical location, the cancer of the gastrointestinal tract fluctuates between the fourth (colorectal cancer) to the fifteenth place (pancreatic cancer) of the cancers worldwide for both sexes, causing a mortality age-standardised rate of between 8.4 (8.9%) and 4.1 (4%) per 100,000 inhabitants [1].

Haemorrhages, intestinal perforation and obstruction are three main complications of patients with gastrointestinal cancer, due to these ~25,000 deaths arise annually in the United States (US) [2–5]. These complications demand a high availability of blood (blood products) for transfusion.

Health institutions specialised in oncology should know which blood components are more required since the oncological patient demands quality and efficiency in their Hemotherapy processes. To be exact, the institutions must emphasise the storage of the most frequent blood products and know what type of neoplasia demands greater transfusions. They must also maintain the stock of blood products to provide quality care.

In Peru, like other low-and-middle income countries, the scientific societies and health institutions do not statement the frequency, the adequate and efficient use and the availability of blood products in patients with gastrointestinal cancer [6–8]. Indeed, inadequate blood transfusion prescriptions have been reported in a National tertiary-care hospital in Lima [9].

Blood transfusions are vital for cancer patients who require these due to the problems with the tumour as well as the physiological complications and the treatment activities. However, the transfusion must be balanced against the potential benefits and risks for oncological patients [10–12].

We aimed to determine gastrointestinal cancer that requires a greater number of transfusions recorded in the blood bank service of the Oncosalud Clinic from 2016 to 2018. Moreover, we determine the most used blood products according to the type of gastrointestinal cancer.

## Methods

This cross-sectional retrospective study was conducted in Type I Hemotherapy and Blood Bank Service of a Peruvian Private Oncological Clinic (TI-HBB-OncoSalud) during 2016–2018. This Service meets the internal demand of the clinic through a cooperation agreement with a Type II Blood Bank to supply blood components to oncological patients requiring transfusion. This study was approved by the Institutional Ethics Committee and the authorisation of the Heads of Departments and Services involved in the study. Further, this study complies with international guidelines for the protection of patient information.

### Patients’ inclusion criteria and data gathering

According to the requirements of the Clinic, we were included patients with gastric cancer was Stage I, II, III and IV (International Code of Diseases (ICD): C16.1, K92.2), pancreatic cancer (ICD: C25.0, C25.9, C25.7), liver and bile duct cancer (ICD: C22, C22.1, C22.9, C22.7, C24.0, C23.X), colon tumour (ICD: C18.2, C18.4, C18.7, C18.9, D12.5, D12.6, D12.9), anal carcinoma (ICD: C21.1) and oesophagus cancer (ICD: C15.9).

We were enrolled patients with requests for care, ≥18 years, patients with preneoplastic, neoplastic or relapse and treatment control alterations and patients who had received a complete transfusion and free of complications [6, 13–15]. We consider >4 transfusions as high-transfusion volume. To collect the data, a previously validated form was used (α-Cronbach = 0.085). The data were collected from the physical and electronic reports of the patients.

### Blood donation and transfusion

The donations were made in compliance with the requirements of the Programa Nacional de Hemoterapía y Banco de Sangre and following the Standardised Operational Procedure (SOP) of the Blood Bank. Quadruple TERUMO bags (Shibuya, Tokyo, Japan) were used during the donation, and registered, fractionated blood products were sent in accordance with the operating needs of the clinic. The haematological values of complete blood count (CBC) of each unit were verified in the Sysmex XS-1000i Hematology Autoanalyzer (Kobe, Japan) according to the requirements of oncological patients. The transfusion was performed in compliance with the international and national guidelines for Good Practices in Blood Transfusion and the OncoSalud Clinic’s SOP [10, 16].

### Data analysis

All the data were coded from the Oncology Patients Control Notebook and the Electronic Transfusion Control System of the Clinic to the collection form and a data matrix in MS-Excel 2010 (Redmond, US) for Windows. Clinical data were included (age, sex, type of origin of neoplasm through the ICD) and laboratory data (type of blood product, CBC, etc.).

We used descriptive statistics (frequency distribution, mean and standard deviation) and non-paired *T*-test and chi-square considering a value of *p* < 0.05 and a confidence interval of 95% as statistically significant. The statistical analysis was performed with IBM SPSS v21.0 (Armonk, US) and BloxPlotR (Rstudio, Tyers and Rappsilber labs, US) for Windows.

## Results

Three thousand and twenty-two patients were enrolled during 2016–2018, of which 163 (5.4%) had gastrointestinal cancer. The mean age of the patients was 67.1 ± 12 years (95% CI: 65.3–68.9). The 80 (49.1%) men did not show significant differences with the 83 (50.9%) women (*p* = 0.178).

**Figure 1.**
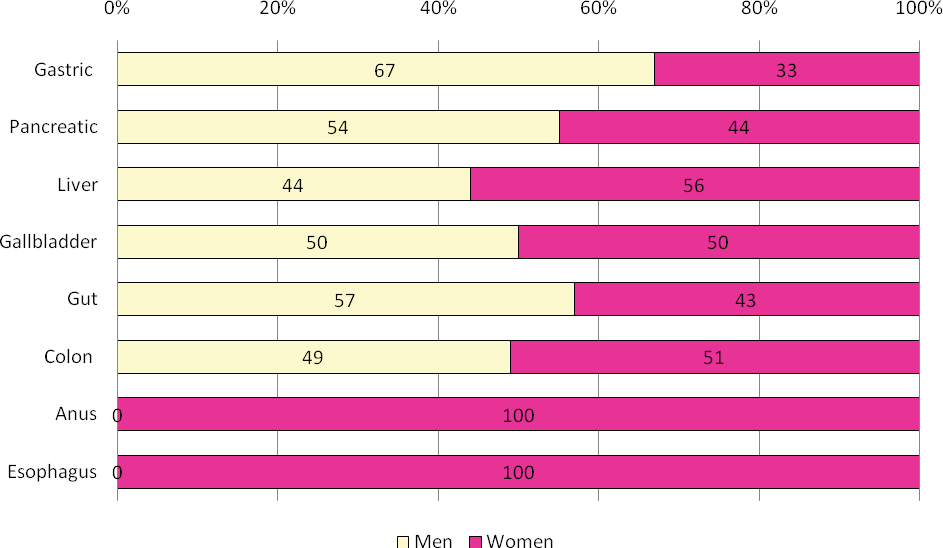
Distribution by sex of all Peruvian patients with a gastrointestinal cancer. We show ~53% of men in patients with gastric (*n* = 6), pancreatic (*n* = 54), liver (*n* = 16), gallbladder (*n* = 6), gut (*n* = 7) and colon (*n* = 68). Conversely, in patients with cancer of anus (*n* = 4) and oesophagus (*n* = 2), the total of patients were women (*p* = 0.002).

According to the type of neoplasia, the most frequent affectation was colon with 41.7% (68 patients) and pancreas with 37.4% (61 patients). Both the malignant tumour of the anal canal (2.4%, four patients) and the malignant tumour of the oesophagus (1.2%, two patients) were the least frequent. Figure 1 shows the distribution of oncologic patients according to sex and type of neoplasia.

Three hundred and four blood products were transfused to patients with gastrointestinal cancer during the study period. The 81.3% (247 units) were red blood cells concentrated, followed by 8.6% (26 units) of fresh-frozen-plasma (FFP) and 6.6% (20 units) of cryoprecipitate (CRYO). The type of cancer that most blood products demanded was colon neoplasia (41.8%), followed by pancreatic cancer (26.3%) and liver cancer (10.9%) (Table 1). Red blood cell concentrate (RBCC) was transfused to all types of cancer, with the exception of oesophageal cancer where only 10 units of CRYO were used for each transfused patient (used mainly in surgery and in the management of pre-surgery complications).

In addition, the average transfusion was 1.8 ± 2.5 units (range: 1–30 units/patient). The maximum number of units transfused was 30 units of RBCC in a patient with colon cancer (75-year-old male, blood group O Rh(D)+ and haemoglobin = 6.2 gr/dL) during surgical management. We determined an association between FFP with gastrointestinal cancer (*p* = 0.001) and with blood groups, (*p* = 0.037) particularly the O Rh (D)+ blood group.

**Figure 2.**
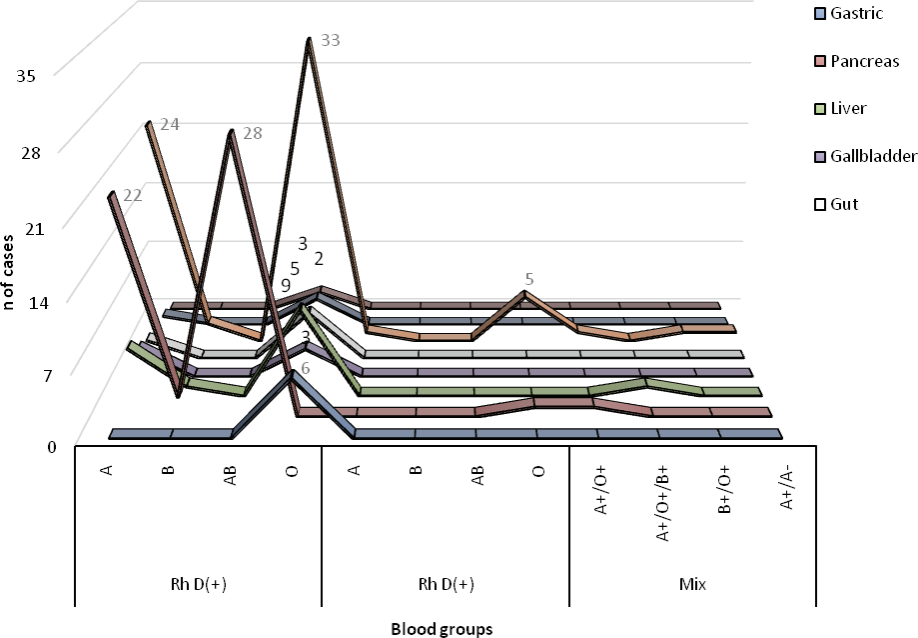
Blood groups of Peruvian patients with gastrointestinal cancer (*n* = 163). The blood group O Rh(D)+ is widely distributed in all types of cancer according to the anatomical site, while the heterogeneous blood groups are only in patients with pancreas, liver and colon cancer.

On the clinical characteristics of patients with gastrointestinal cancer, ~55% of patients were O Rh(D)+ followed by blood group A Rh (D)+ (35%). In five patients, we were observed heterogeneity (poly-transfused) in the characterisation of blood groups due to previous transfusions (Figure 2). Seven (4.3%) patients had a Rh (D) negative blood group (A and O), showing a significant difference compared to Rh (D) positive patients (*p* = 0.001).

Finally, the distributions of the origin of the patients are shown in Table 2. One hundred and three patients (63.2%) derived from hospitalisation followed by 28 patients (17.2%) from the Clinic’s emergency area. Patients with oesophageal cancer (1.23%) derived only from Critical Intensive Unit and Emergency, while patients with colon cancer (41.7%), liver (9.8%) and pancreas (33.1%) derived from the four areas (Table 2). We did not find a significant correlation between age (*p* = 0.248), sex (*p* = 0.306) and blood group (*p* = 0.071) with blood products.

## Discussion

We detected that >78% of blood products were transfused to patients with colon, pancreas and liver cancer. These transfusions were mostly of RBCC (~80%), and in oesophageal cancer, the only CRYO was transfused. Our findings also suggest that more than 300 blood products were transfused in 163 patients with gastrointestinal cancer, where >50% were men, with blood group O Rh (D)+, and from hospitalisation areas of the clinic.

The main strength of the study is that we developed the first study on the impact of allogenic blood transfusion in Peruvian patients with gastrointestinal cancer. Due to the lack of current information on this topic, we emphasise its importance and provide useful information for the management of donations/transfusion by the Blood Banks. Another important aspect is that we determine that Hospitalised area (HA) demanded the most transfusions. To best meet the blood transfusion needs and improve the service quality of the blood bank, it is necessary to know which area or department of the clinic needs more blood derivatives.

Complications in patients with the oncological disease are usually diverse. In addition to those mentioned above, there are post-surgical complications [17, 18] and metastases that certain cancers develop [19]. It has been proven for more than 35 years ago that blood transfusions can be harmful in patients with colon cancer [11, 12, 20], ovarian cancer [21], hepatocellular carcinoma [22], gastric cancer [10], amongst others.

In this study, we did not register adverse effects after the transfusion. In part, the mortality rate in these patients could be due to the complications of advanced cancer and neoplastic components (i.e. immunity, nutrition), which could also be due to unjustified transfusions, as has happened in several hospitals in Lima [9, 23]. The patients who received the most transfusions are those who have the highest risk of gastrointestinal bleeding/coagulopathies.

Recently, a Peruvian retrospective study showed that critical care areas (i.e. Critical Intensive Unit (CIU)) request more transfusions of blood products [24]. The reason for these requests was mainly due to anaemia (>30%), and the most requested and transfused blood products were the red blood cells concentrated (~60%), followed by the FFP with 25%. Our findings agree with this previous study.

The complications of several cancers have generated high costs in the US [25]. The transfusion constitutes the medical practice to safeguard the life of the patients during the surgery, to avoid post-surgical complications and to attend the requirements that the patients with gastrointestinal cancer demand [26]. As part of the quality requirements of public and private Hemotherapy and Blood Bank centres, there is the imperative to know the needs and health realities of its users and to effectively meet all the requirements of cancer patients. Therefore, the Blood Banks must possess stocks of the most demanded blood products and a constant flow of high-quality pathogen-free donations [27].

In Peru, it has been described that ≥70% of the Peruvian population has group O Rh (D) +, followed by blood group A Rh(D)+ (≥18%) and B Rh(D)+ (≥8%) [28]. Our findings showed that the most frequent blood groups of patients with gastrointestinal cancer agree with these estimates. Likewise, the blood products most transfused were the red blood cells concentrated and the FFP (Table 1). Patients with more frequent Blood Groups receive the freshest blood or had it available versus the least frequent blood that was delayed (time) or not available.

Regardless of the type of cancer [2, 22, 29] or other diseases [30–32], the red blood cells concentrated and the FFP are the main blood products transfused worldwide. In both cases, the Blood Banks must ensure and organise the cyclical viability of these products.

This study has limitations. The first limitation is the number of patients enrolled in the study. Given the nature and period of study, we were unable to include more patients with gastrointestinal cancer who required transfusions. Second, we did not have access to the follow-up of the transfused patients; hence we could not evaluate the large post-transfusion complications of each blood product. Finally, data on patient follow-up has not been available because the patients come from different clinics in Lima (capital of Peru) and regions. By not having a standardised system, patients are lost during follow-up or are treated later in other health centres. For this reason, we were unable to know the impact of transfusions on disease-free survival, overall survival and quality of life. Further longitudinal investigation is also required to determine and differentiate transfusion adverse reactions related to blood products and neoplasia.

## Conclusion

This earliest Peruvian study showed that patients with gastrointestinal cancer require a large number of transfusions of red blood cells concentrated and the FFP. Likewise, we showed that cancer of the colon, pancreas and liver demanded more than 75% of blood products.

## Conflicts of interest

The authors declare that there are no conflicts of interest.

## Authors’ contributions

All the authors have made important intellectual contributions to this article.

## Funding

The authors did not receive specific funding for this work.

## Figures and Tables

**Table 1.** Amount of blood products transfusing to Peruvian patients with gastrointestinal cancer (*n* = 163).

Type of cancer	Blood products				Total
	RBCC	PQ	FFP	CRYO	
Gastric (*n* = 6)^a^	9 (3)	2 (0.7)	4 (1.3)	—^c^	15 (4.9)
Pancreas (*n* = 54)	75 (24.7)	1 (0.3)	4 (1.3)	—	80 (26.3)
Liver (*n* = 16)^b^	22 (7.2)	2 (0.7)	9 (3)	—	33 (10.9)
Gallbladder (*n* = 6)	5 (1.6)	—	2 (0.7)	—	7 (2.3)
Gut (*n* = 7)	9 (3)	2 (0.7)	4 (1.3)	—	15 (4.9)
Colon (*n* = 68)	121 (39.8)	4 (1.3)	2 (0.7)	—	127 (41.8)
Anus (*n* = 4)	6 (2)	—	1 (0.3)	—	7 (2.3)
Oesophagus (*n* = 2)	—	—	—	20 (6.6)	20 (6.6)
Total	247 (81.3)	11 (3.6)	26 (8.6)	20 (6.6)	304 (100)

a Four units of RBCC and four units of FFP were transfused in one patient

b Three units of RBCC and one unit of FFP were transfused in a patient

c Not transfusedRBCC, Red blood cell concentrate; PQ, Platelets; FFP, Fresh frozen plasma; CRYO, Cryoprecipitate

**Table 2.** Distribution of Peruvian patients with gastrointestinal cancer according to the Clinic’s place of origin of the four areas (*n* = 163).

Type of cancer	Area of provenance				Total
	HA	OR	CIU	ER	
Gastric	1 (0.61)	1 (0.61)	4 (2.45)	—^a^	6 (3.7)
Pancreas	35 (21.5)	2 (1.23)	2 (1.23)	15 (9.2)	54 (33.1)
Liver	7 (4.3)	7 (4.3)	1 (0.61)	1 (0.61)	16 (9.8)
Gallbladder	3 (1.8)	3 (1.8)	—	—	6 (3.7)
Gut	2 (1.23)	1 (0.61)	4 (2.45)	—	7 (4.3)
Colon	52 (31.9)	4 (2.45)	2 (1.23)	10 (6.13)	68 (41.7)
Anus	3 (1.84)	—	—	1 (0.61)	4 (2.45)
Oesophagus	—	—	1 (0.61)	1 (0.61)	2 (1.23)
Total	103 (63.2)	18 (11)	14 (8.6)	28 (17.2)	163 (100)

a Without cases

HA, Hospitalised area; OR, Operations room; CIU, Critical intensive unit; ER, Emergency
